# Optimization of mNeonGreen for *Homo sapiens* increases its fluorescent intensity in mammalian cells

**DOI:** 10.1371/journal.pone.0191108

**Published:** 2018-01-17

**Authors:** Emiko Tanida-Miyake, Masato Koike, Yasuo Uchiyama, Isei Tanida

**Affiliations:** 1 Department of Cell Biology and Neuroscience, Juntendo University School of Medicine, Tokyo, Japan; 2 Department of Gastroenterology, Machida Municipal Hospital, Tokyo, Japan; 3 Department of Cellular and Molecular Neuropathology, Juntendo University School of Medicine, Tokyo, Japan; Osaka University, JAPAN

## Abstract

Green fluorescent protein (GFP) is tremendously useful for investigating many cellular and intracellular events. The monomeric GFP mNeonGreen is about 3- to 5-times brighter than GFP and monomeric enhanced GFP and shows high photostability. The maturation half-time of mNeonGreen is about 3-fold faster than that of monomeric enhanced GFP. However, the cDNA sequence encoding mNeonGreen contains some codons that are rarely used in *Homo sapiens*. For better expression of mNeonGreen in human cells, we synthesized a human-optimized cDNA encoding mNeonGreen and generated an expression plasmid for humanized mNeonGreen under the control of the cytomegalovirus promoter. The resultant plasmid was introduced into HEK293 cells. The fluorescent intensity of humanized mNeonGreen was about 1.4-fold higher than that of the original mNeonGreen. The humanized mNeonGreen with a mitochondria-targeting signal showed mitochondrial distribution of mNeonGreen. We further generated an expression vector of humanized mNeonGreen with 3xFLAG tags at its carboxyl terminus as these tags are useful for immunological analyses. The 3xFLAG-tagged mNeonGreen was recognized well with an anti-FLAG-M2 antibody. These plasmids for the expression of humanized mNeonGreen and mNeonGreen-3xFLAG are useful tools for biological studies in mammalian cells using mNeonGreen.

## Introduction

Green fluorescent protein (GFP) was first isolated from *Aequorea victoria* jelly fish, and GFP-variants, like monomeric enhanced GFP (mEGFP) [1], are useful tools for investigating cellular and intracellular events, including organelle morphology, intracellular localization of proteins, intracellular Ca^2+^ concentration, pH monitoring, and protein-protein interactions [2–8]. The main advantage of GFP is its fluorescent intensity and stability in cells. Recently, one of brightest GFPs, mNeonGreen, was derived from *Branchiostoma lanceolatum* (European lancelet) and has several advantages over EGFP and mEGFP [1, 3–5, 9, 10]. mNeonGreen is a monomeric protein that is about 3- to 5-fold brighter than GFP and EGFP, and its maturation time is about 3-fold shorter than that of EGFP [2, 3]. These properties indicate mNeonGreen is a better fluorescent tool than EGFP and mEGFP to investigate biological functions [1, 2, 10].

To utilize mNeonGreen in mammalian cells more effectively, it is important to obtain efficient expression. Previously, mNeonGreen cDNA was found to contain at least seven codons used rarely in *H*. *sapiens* [3]. Therefore, we hypothesized that expression of mNeonGreen in mammalian cells would improve upon optimization of its codons for *H*. *sapiens*. Another disadvantage of mNeonGreen versus mEGFP and EGFP is that there are few antibodies available for mNeonGreen recognition. While there are many commercially available antibodies for GFP and its other variants, they cannot be used to detect mNeonGreen due to the low amino acid sequence homology between mNeonGreen and mEGFP (Table 1). Therefore, we also generated a plasmid that would help optimize expression of 3xFLAG-tagged, humanized mNeonGreen [11]. These improvements will be very useful for analyzing biological events using this protein in mammalian cells.

**Table 1:** Comparison of the amino acid sequence of mNeonGreen with that of EGFP using Clustal-W alignment.

mNeonGreen	1	M	V	S	K	G	E	E	D	N	M	A	S	L	P	A	T	H	E	L	H	I	F	G	S	I	N	G	V	D	F	D	M	V	G	Q	G	T	G	N	P
EGFP	1	M	V	S	K	G	E	E	L	F	T	G	V	V	P	I	L	V	E	L	D	-	-	G	D	V	N	G	H	K	F	S	V	S	G	E	G	E	G	D	A
		*	*	*	*	*	*	*						.	*				*	*				*		.	*	*			*		.		*	.	*		*		
mNeonGreen	41	N	D	G	Y	E	E	L	N	L	K	S	T	K	G	D	L	Q	F	S	P	W	I	L	V	P	H	I	G	Y	G	F	H	Q	Y	L	P	Y	P	D	G
EGFP	38	T	Y	G	K	L	T	L	K	F	I	C	T	T	G	K	L	P	V	P	W	P	T	L	V	T	T	L	T	Y	G	V	Q	C	F	S	R	Y	P	D	H
		.		*				*				.	*		*		*							*	*			.		*	*		.		.			*	*	*	
mNeonGreen	81	M	S	P	-	-	-	F	Q	A	A	M	V	D	G	S	G	Y	Q	V	H	R	T	M	Q	F	E	D	G	A	S	L	T	V	N	Y	R	Y	T	Y	E
EGFP	70	M	K	Q	H	D	F	F	K	S	A	M	P	E	G	-	-	Y	V	Q	E	R	T	I	F	F	K	D	D	G	N	Y	K	T	R	A	E	V	K	F	E
		*						*	.	.	*	*		.	*			*				*	*	.		*		*												.	*
mNeonGreen	118	G	S	H	I	K	G	E	A	Q	V	K	G	T	G	F	P	A	D	G	P	V	M	T	N	S	L	T	A	A	D	W	C	R	S	K	K	T	Y	P	N
EGFP	117	G	D	T	L	V	N	R	I	E	L	K	G	I	D	F	K	E	D	G	N	I	L	G	H	K	L	E	Y	N	Y	N	S	H	N	V	Y	I	M	A	D
		*			.		.			.	.	*	*			*			*	*		.	.		.		*						.	.							
mNeonGreen	158	D	K	-	-	T	I	I	S	T	F	K	W	S	Y	T	T	G	N	G	K	R	Y	R	S	T	A	R	T	T	Y	T	F	A	K	P	M	A	A	N	-
EGFP	157	K	Q	K	N	G	I	K	V	N	F	K	I	R	H	N	I	E	D	G	S	-	-	-	V	Q	L	A	D	H	Y	Q	Q	N	T	P	I	G	D	G	P
				.			*			.	*	*				.				*											*					*	.				.
mNeonGreen	195	Y	L	K	N	Q	P	M	Y	V	F	R	K	T	E	L	K	H	S	K	T	E	L	N	-	-	-	-	F	K	E	W	Q	K	A	F	T	D	V	M	G
EGFP	194	V	L	L	P	D	N	H	Y	L	S	T	Q	S	A	L	S	K	D	P	N	E	K	R	D	H	M	V	L	L	E	F	V	T	A	A	G	I	T	L	G
			*						*	.			.	.		*		.			.	*									*				*					.	*
mNeonGreen	231	M	D	E	L	Y	K																																		
EGFP 234 M	234	M	D	E	L	Y	K																																		
		*	*	*	*	*	*																																		

mNeonGreen had low sequence homology with EGFP (27% identity, 14% similarity).

EGFP, enhanced green fluorescent protein.

## Materials and methods

### Cells, media, materials, and antibodies

HEK293 and COS1 cells were obtained from the American Type Culture Collection, and cultured in Dulbecco’s Modified Eagle’s Medium (Wako, 045–30285) containing 10% fetal calf serum (JRH Biosciences/Sigma-Aldrich, 12603C). The mouse monoclonal antibody, clone M2, against the FLAG peptide (DYKDDDDK; F1804) was purchased from Sigma-Aldrich, and the mouse monoclonal antibody against glyceraldehyde-3-phosphate dehydrogenase was from Abcam (ab8245). Protein concentrations were determined using the bicinchoninic acid protein assay (Pierce, 23225). FuGENE HD transfection reagent was used to introduce the plasmid into cells (Promega, E2311). mCherry2-C1 was a gift from Dr. Michael Davidson (Addgene plasmid # 54563), and pLL7.0: Venus-iLID-Mito (From ActA) was from Dr. Brian Kuhlman (Addgene plasmid # 60413) [12].

### DNA synthesis and construction of plasmids for expression of humanized mNeonGreen and mNeonGreen-3xFLAG

**Fig 1:**
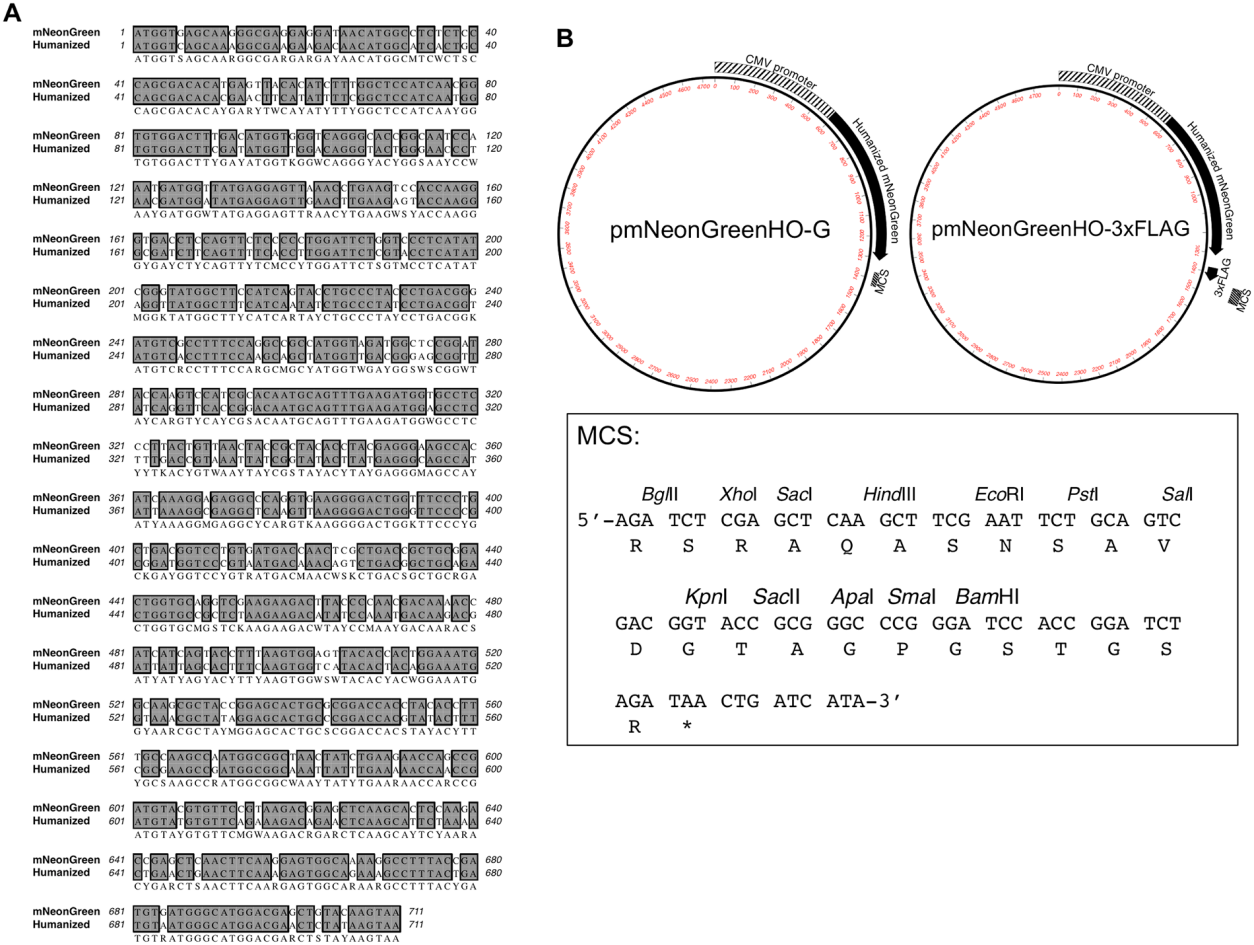
DNA sequence and plasmid maps of mNeonGreen. **(A) Comparison of the DNA sequence of humanized mNeonGreen with that of the original one.** A pairwise alignment of two DNA sequences of humanized (GenBank Accession No. LC279210) and original mNeonGreen (GenBank Accession No. KC295282) was performed using a CLUSTAL W program (http://clustalw.ddbj.nig.ac.jp/). **(B) Plasmid maps for the expression of humanized mNeonGreen and mNeonGreen-3xFLAG.** Humanized mNeonGreen cDNA with a triple Gly-Gly-Gly-Ser linker was inserted into the *Nhe*I-*Bgl*II site of pAcGFP-C1 after removing AcGFP cDNA to create pmNeonGreenHO-G. Humanized mNeonGreen-3xFLAG cDNA was inserted into the *Nhe*I-*Bgl*II site of pAcGFP-C1 after removing AcGFP cDNA to create pmNeonGreenHO-3xFLAG. MCS, multicloning sites; CMV, cytomegalovirus.

mNeonGreen amino acid sequence (GenBank Accession No. AGG56535.1) codon optimization for *H*. *sapiens* was performed using MacVector software based on the codon usage database on the Kazusa DNA Research Institute website (http://www.kazusa.or.jp/codon/cgi-bin/showcodon.cgi?species=9606). The resultant optimized DNA fragment was synthesized by Integrated DNA Technologies with a linker sequence (5’-AAAAAAGCTAGCGCCACC-3’) before the start codon and another (5’-AAGTCCGGAACTAGTTTTTT-3’) before the stop codon. The synthesized DNA fragment was digested by restriction enzymes *Nhe*I and *Bsp*EI, and a *Nhe*I-*Bsp*EI site was introduced to pAcGFP-G; pAcGFP-G is similar to pAcGFP-C1 and contains a triple Gly-Gly-Gly-Ser linker sequence at the C-terminus before its multicloning site. The resultant plasmid was designated as pmNeonGreenHO-G (Fig 1). For the expression of original mNeonGreen, a DNA fragment of mNeonGreen (GenBank Accession No. AGG56535.1) was synthesized by Integrated DNA Technologies with a linker sequence (5’-AAAAAAGCTAGCGCCACC-3’) before the start codon and another (5’-AAGTCCGGAACTAGTTTTTT-3’) before the stop codon. The plasmid used for expression of original mNeonGreen (pmNeonGreen-G) was constructed using the same strategy as for humanized mNeonGreen above.

For the expression of mitochondria-localized humanized mNeonGreen protein, we amplified a DNA fragment encoding a mitochondrial targeting signal of *ActA* gene of *Listeria monocytogenes* derived from pLL7.0: Venus-iLID-Mito (From ActA) using a mtSig-ActA-Bgl2-F primer (5’-CCCAGATCTAAACTAATTGCTAAAAGTGCAGAA-3’) and a mtSig-ActA-ST-SalI-Rv primer (5imCCCGTCGACTTAATTATTTTTTCTTAATTGAAT-3’)[12]. The amplified DNA fragment was digested with *Bgl*II and *Sal*I. The *Bgl*II-*Sal*I DNA fragment was introduced into the *Bgl*II-*Sal*I site of pmNeonGreenHO-G. The resultant plasmid was designated as pmNeonGreenHO-mito.

For the expression of C-terminal 3xFLAG-tagged, humanized mNeonGreen, a DNA fragment encoding 2xSTREP-3xFLAG (5’-GCTAGCGCCACCATGTGGAGCCACCCGCAGTTCGAGAAAGGTGGAGGTTCCGGAGGTGGATCGGGAGGTGGATCGTGGAGCCACCCGCAGTTCGAAAAAACTAGTCGGGCTGACTACAAAGACCATGACGGTGATTATAAAGATCATGACATCGACTACAAGGATGACGATGACAAGAGATCTTAGGCGGCCGCTCGAGTCTAGAGGGCCCG-3’) was synthesized. The DNA fragment was digested by *Bsp*EI-*Bgl*II and inserted into a *Bsp*EI-*Bgl*II site. The resultant plasmid used for the expression of mNeonGreen-3xFLAG was designated as pmNeonGreenHO-3xFLAG.

### Immunoblotting

Cells were washed twice in phosphate-buffered saline and lysed in lysis buffer (10 mM sodium phosphate [pH 7.2], 150 mM NaCl, and 1% sodium dodecyl sulfate [SDS]) containing a protease-inhibitor cocktail (04080–24, Nacalai Tesque). Lysate proteins (10 μg) were separated by SDS-polyacrylamide gel electrophoresis. After transferring the proteins to a polyvinylidine difluoride membrane using a Trans-Blot SD transfer cell (Bio-Rad, 170–3940), mNeonGreen-3xFLAG and glyceraldehyde-3-phosphate dehydrogenase were recognized using the appropriate antibodies and developed with a chemiluminescent method according to standard protocols using SuperSignal West Dura Extended Duration Substrate (Pierce, 34075).

### Fluorescence microscopy

Cells were fixed in fixation solution (4% paraformaldehyde in phosphate-buffered saline) at room temperature for 5 min, and fluorescence was monitored using a BZ-X700 microscope (Keyence, Tokyo, Japan).

### Analyses of fluorescence intensity of humanized mNeonGreen

The plasmids, pmNeonGreen-G or pmNeonGreenHO-G was transfected into HEK293 cells. As a control of transfection efficiency, mCherry2-C1 was employed. At 48 h after transfection, the fluorescent intensity of mNeonGreen and mCherry in 293 cells was analyzed using a 2300 EnSpire multimode reader (PerkinElmer, Massachusettes).

## Results and discussion

### Increased fluorescent intensity of humanized mNeonGreen in mammalian cells

mNeonGreen is one of brightest GFPs derived from *Branchiostoma lanceolatum*. To investigate whether the reported cDNA sequence of mNeonGreen (GenBank Accession No. KC295282) is suitable for expression in human cells, we analyzed its codon usage with the Kazusa DNA Research Institute website (http://www.kazusa.or.jp/codon/cgi-bin/showcodon.cgi?species=9606) [Table 2].

**Table 2:** *Homo sapiens* codon usage.

codon	AA	FRT	codon	AA	FRT	codon	AA	FRT	codon	AA	FRT
UUU	F	17.6	UCU	S	15.2	UAU	Y	12.2	UGU	C	10.6
UUC	F	20.3	UCC	S	17.7	UAC	Y	15.3	UGC	C	12.6
UUA	L	7.7	UCA	S	12.2	UAA	*	1	UGA	*	1.6
UUG	L	12.9	UCG	S	4.4	UAG	*	0.8	UGG	W	13.2
CUU	L	13.2	CCU	P	17.5	CAU	H	10.9	CGU	R	4.5
CUC	L	19.6	CCC	P	19.8	CAC	H	15.1	CGC	R	10.4
CUA	L	7.2	CCA	P	16.9	CAA	Q	12.3	CGA	R	6.2
CUG	L	39.6	CCG	P	6.9	CAG	Q	34.2	CGG	R	11.4
AUU	I	16	ACU	T	13.1	AAU	N	17	AGU	S	12.1
AUC	I	20.8	ACC	T	18.9	AAC	N	19.1	AGC	S	19.5
AUA	I	7.5	ACA	T	15.1	AAA	K	24.4	AGA	R	12.2
AUG	M	22	ACG	T	6.1	AAG	K	31.9	AGG	R	12
GUU	V	11	GCU	A	18.4	GAU	D	21.8	GGU	G	10.8
GUC	V	14.5	GCC	A	27.7	GAC	D	25.1	GGC	G	22.2
GUA	V	7.1	GCA	A	15.8	GAA	E	29	GGA	G	16.5
GUG	V	28.1	GCG	A	7.4	GAG	E	39.6	GGG	G	16.5

40662582 codons of *Homo sapiens* were analyzed with the Kazusa DNA Research Institute website (http://www.kazusa.or.jp/codon/).

AA, single letter amino acid code; FPT, frequency per 1000 codons.

The original mNeonGreen mRNA sequence was found to contain some codons that are rarely used in *H*. *sapiens*, especially two UUA (7.7 frequency per 1000 codons), three UCG (4.4 frequency per 1000 codon), one CCG (6.9 frequency per 1000 codons) and one CGU (4.5 frequency per 1000 codons) [Table 3]. Therefore, we concluded that optimization of the mNeonGreen codon sequence for *H*. *sapiens* would improve its expression and thereby, its fluorescent intensity, in mammalian cells.

**Table 3:** Codon usage in original mNeonGreen.

codon	FRT	n	codon	FRT	n	codon	FRT	n	codon	FRT	n
UUU	25.4	6	UCU	4.2	1	UAU	12.7	3	UGU	0.0	0
UUC	25.4	6	UCC	25.3	6	UAC	50.6	12	UGC	4.2	1
UUA	8.4	2	UCA	0.0	0	UAA	4.2	1	UGA	0.0	0
UUG	0.0	0	UCG	12.7	3	UAG	0.0	0	UGG	16.9	4
CUU	4.2	1	CCU	21.1	5	CAU	16.9	4	CGU	4.2	1
CUC	16.9	4	CCC	12.7	3	CAC	12.7	3	CGC	12.7	3
CUA	0.0	0	CCA	12.7	3	CAA	8.4	2	CGA	0.0	0
CUG	25.3	6	CCG	4.2	1	CAG	29.5	7	CGG	8.4	2
AUU	4.2	1	ACU	21.1	5	AAU	12.7	3	AGU	8.4	2
AUC	25.3	6	ACC	54.9	13	AAC	38.0	9	AGC	12.7	3
AUA	0.0	0	ACA	8.4	2	AAA	8.4	2	AGA	0.0	0
AUG	46.4	11	ACG	4.2	1	AAG	67.5	16	AGG	4.2	1
GUU	4.2	1	GCU	12.7	3	GAU	21.1	5	GGU	29.5	7
GUC	8.4	2	GCC	29.5	7	GAC	33.8	8	GGC	33.8	8
GUA	4.2	1	GCA	0.0	0	GAA	4.2	1	GGA	16.9	4
GUG	29.5	7	GCG	16.9	4	GAG	46.4	11	GGG	12.7	3

FPT, frequency per 1000 codons; n, number of codons used in mNeonGreen cDNA

**Fig 2:**
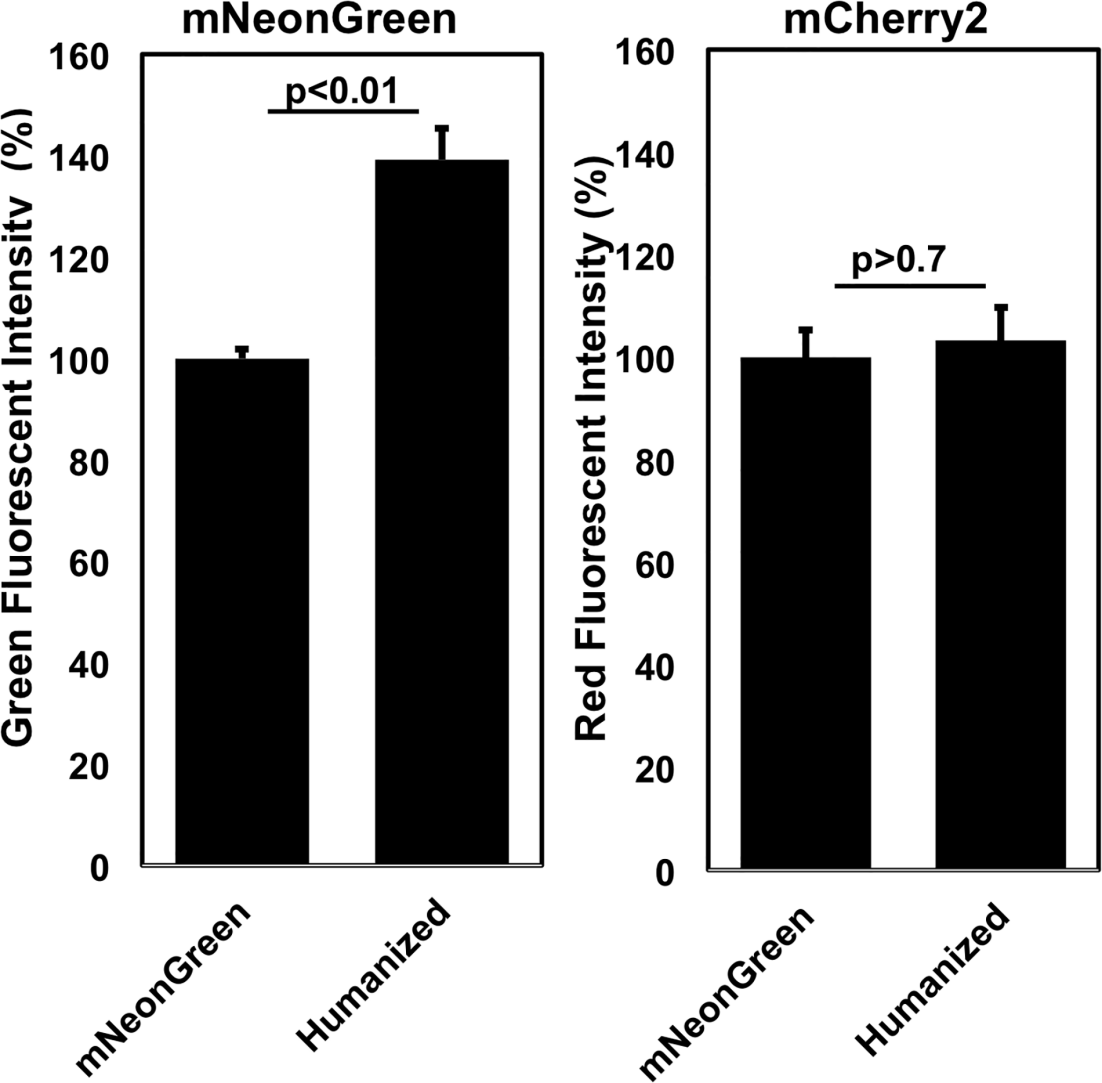
Original and humanized mNeonGreen fluorescence in HEK293. Plasmids designed for expression of original or humanized mNeonGreen were transfected into HEK293 cells. As a control of transfection efficiency, mCherry2-C1 was employed. Fluorescent intensity of mNeonGreen and mCherry2 was obtained 48 h after transfection using a 2300 EnSpire multimode reader. The data were analyzed with a Welch’s *t*-test; p < 0.01 in green fluorescent intensity of **mNeonGreen**; p > 0.7 in red fluorescent intensity of **mCherry2**. Graphs show the relative fluorescent intensity of both fluorescent proteins (%).

Accordingly, we changed 51 out of 236 codons within mNeonGreen (GenBank Accession No. KC295282) to more frequently used codons in *H*. *sapiens*, synthesized a DNA fragment encoding humanized mNeonGreen (GenBank Accession No. LC279210), and generated our expression pmNeonGreenHO-G (Fig 1). The resultant plasmid was transfected into HEK293 cells, and the fluorescent intensity of mNeonGreen in the cells 24 h after transfection was examined. As a control for transfection efficiency, mCherry2-C1 was employed. The fluorescent intensity of humanized mNeonGreen was significantly increased (1.39 ± 0.06-fold, p<0.01) compared with that of original mNeonGreen, while there is no significant difference of that of mCherry2 (Fig 2). This increase was obtained by substituting codons within original mNeonGreen cDNA that are rarely used in humans to more highly used codons. Especially, the codons, UUA, UCG, CGU, and CCG, are rarely used in *Mus musculus* and *Rattus rattus*, therefore the fluorescent intensity of humanized mNeonGreen will be increased in mouse and rat cell lines.

### Humanized mNeonGreen with a mitochondria-targeting signal was showed mitochondria-distribution

**Fig 3:**
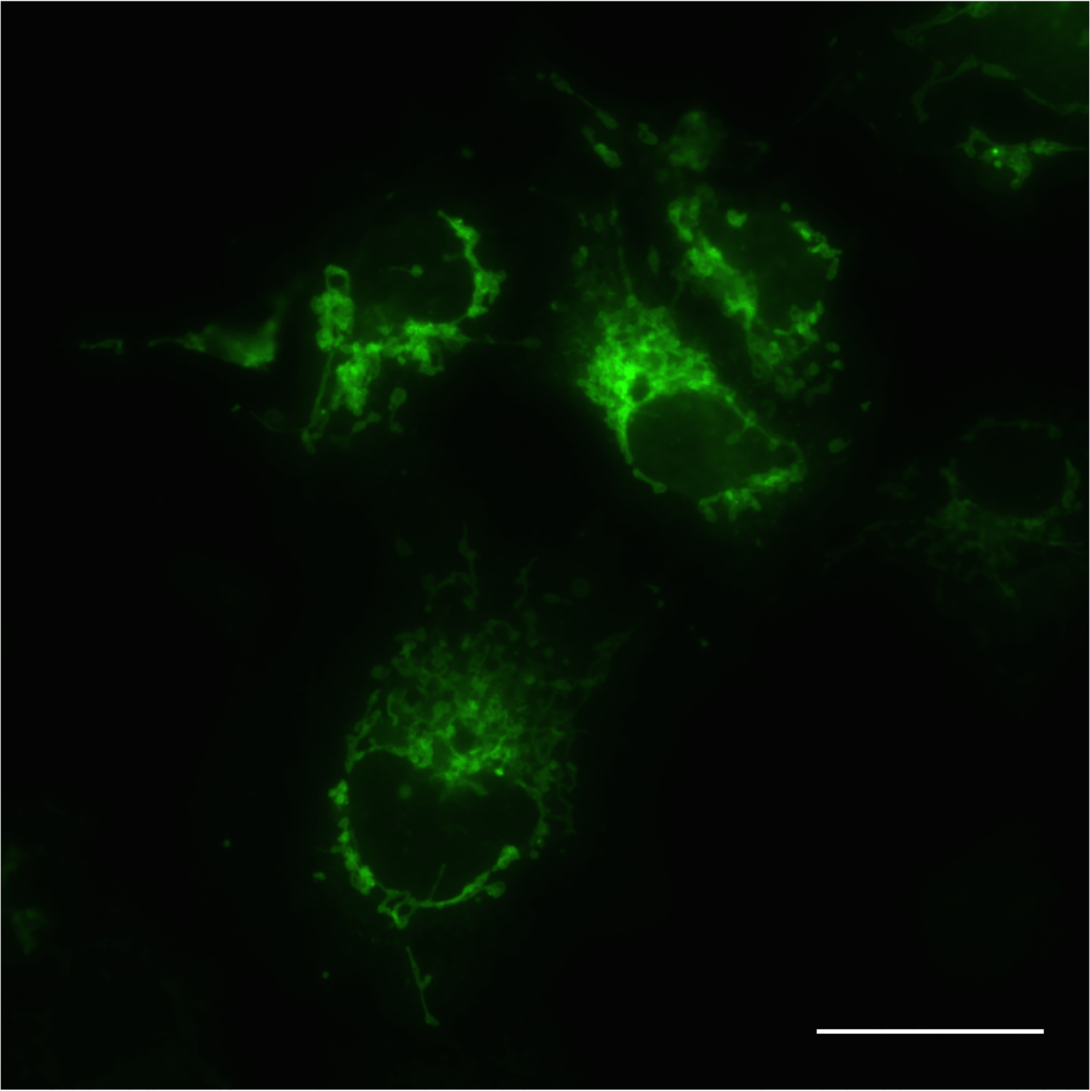
Mitochondrial distribution of humanized mNeonGreen tagged with a mitochondria-targeting signal. The plasmid pmNeonGreen-mito was transfected into COS1 cells. 48 h after transfection, the green fluorescent images in the cells were monitored using a BZ-X700 microscope. Bar indicates 50 μm.

To investigate whether the pmNeonGreenHO-G is suitable for monitoring intracellular organelle, we generated a plasmid, pmNeonGreenHO-mito, for the expression of a mNeonGreen with a mitochondria-targeting signal. This plasmid was transfected into COS1 cells, and its green fluorescence in the cells were investigated. As shown in Fig 3, the humanized mNeonGreen with a mitochondria-targeting signal showed a representative distribution of mitochondria. Therefore, at least, the pmNeonGreenHO-G is available to monitor mitochondria morphology. The original mNeonGreen is available to investigate the other organelle-morphology [3].

Considering the codon-optimization for *Homo sapiens* leads to no change of amino acid sequence of mNeonGreen protein itself, this plasmid can be used for monitoring the intracellular distribution of a certain protein as a fusion protein

### Generation of mNeonGreen-3xFLAG expression plasmid

**Fig 4:**
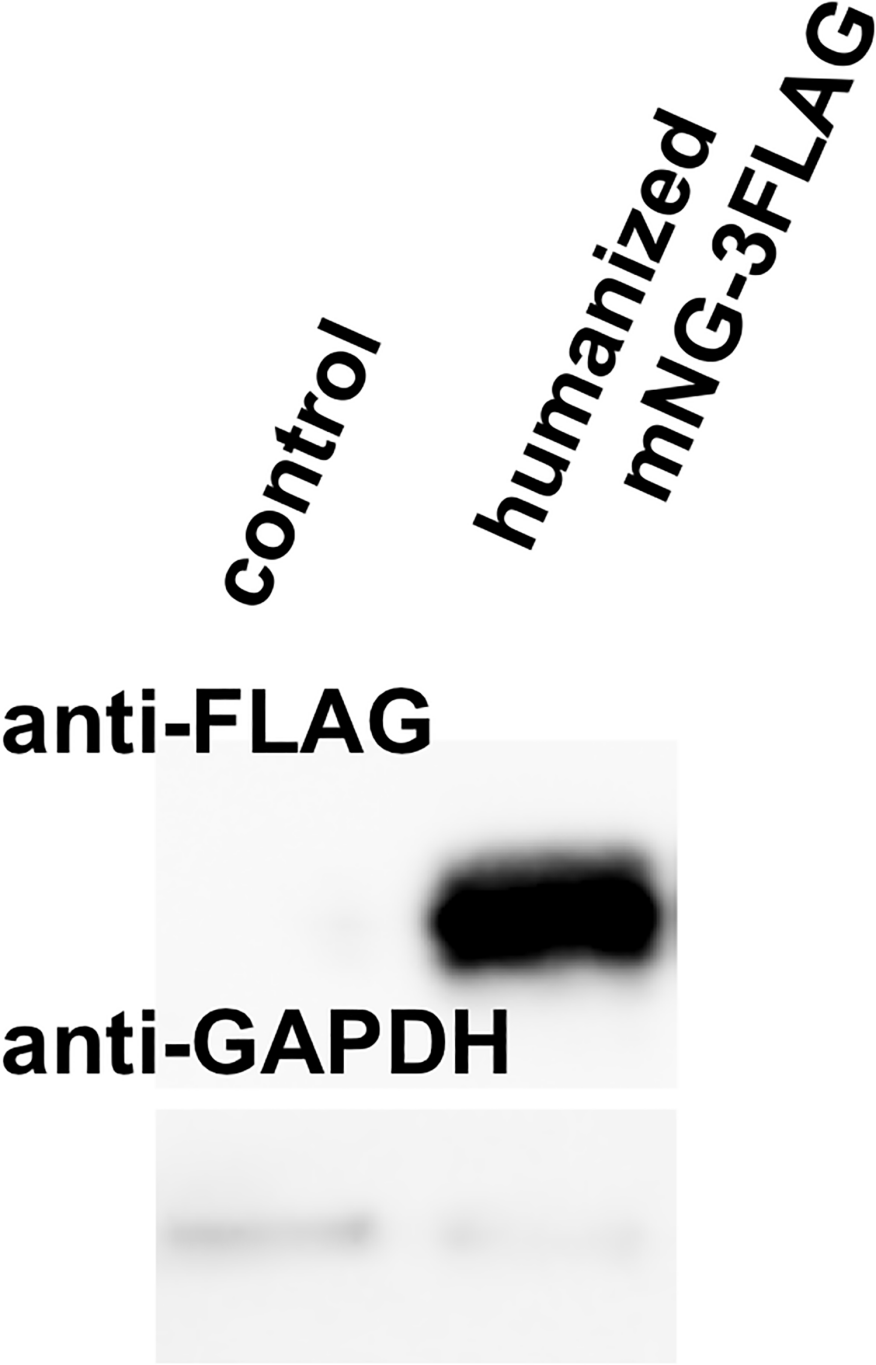
Expression of humanized mNeonGreen-3xFLAG in HEK293 cells. A pmNeonGreenHO-3xFLAG plasmid for expression of humanized mNeonGreen-3xFLAG was introduced into HEK293 cells. Cells were lysed 24 h after transfection, and total proteins in the lysate were separated on 12.5% SDS-polyacrylamide gels. The mNeonGreen-3xFLAG fusion protein was detected with an anti-FLAG M2 antibody. As a loading control, glyceraldehyde-3-phosphate dehydrogenase (GAPDH) was recognized using anti-glyceraldehyde-3-phosphate dehydrogenase antibody.

Clustal-W analyses revealed the amino acid sequence of mNeonGreen has low homology with that of EGFP (27% identity, 14% similarity; Table 1), indicating most commercially available anti-GFP antibodies will not react with mNeonGreen. To solve this problem, we generated the pmNeonGreenHO-3xFLAG expression plasmid (Fig 1). The plasmid was introduced into HEK293 cells and lysates prepared 24 h after transfection. Total proteins were separated by SDS-polyacrylamide gel electrophoresis, and the mNeonGreen-3xFLAG protein was recognized with an anti-FLAG-M2 antibody (Fig 4). As expected, an approximately 33 kDa band corresponding to mNeonGreen-3xFLAG was recognized. FLAG tags are one of the most employed epitopes for biochemical and cellular biology analyses. In conclusion, our pmNeonGreenHO-3xFLAG plasmid and mNeonGreen-3xFLAG fusion protein system will provide certain advantages to biochemical studies in mammalian cells.

## Supporting information

S1 Fig The original fluorescent images of mNeonGreen used in Fig 3. (TIF)

S2 Fig The original immunoblotting images in Fig 4. (**A**) The original immunoblotting data of Humanized mNeonGreen with 3xFlag tag using mouse monoclonal anti-Flag-M2 antibody. The lysate of cells transfected into pmNeonGreenHO-3xFLAG (**mNeonGreenHO-3xFlag**) or a control plasmid (**CON**) was separated on SDS-PAGE, and Flag-tagged proteins and GAPDH were recognized. From the left, molecular weight marker (Mk, lanes 1 and 7), negative control (CON) (lanes 2 and 8), mNeonGreen-3xFlag (lanes 3–6 and 9–12). (**B**) The original data of Humanized mNeonGreen with 3xFlag tag using a rabbit polyclonal anti-glyceraldehyde-3-phosphate dehydrogenase (GAPDH) antibody. As a loading control, GAPDH in the lysate was recognized after removing anti-Flag-M2 antibody.
(TIFF)

## Abbreviations

3xFLAG: triple repeat of FLAG-tag
EGFP: enhanced GFP
GFP: green fluorescent protein
mEGFP: monomeric enhanced GFP
p: the p value of Welch’s t-test
