# Supplementary figures and images for: Optimization of mNeonGreen for Homo sapiens increases its fluorescent intensity in mammalian cells
Source: PLoS One. 2018 Jan 17;13(1):e0191108. doi: 10.1371/journal.pone.0191108 (PMC5771595; doi:10.1371/journal.pone.0191108)

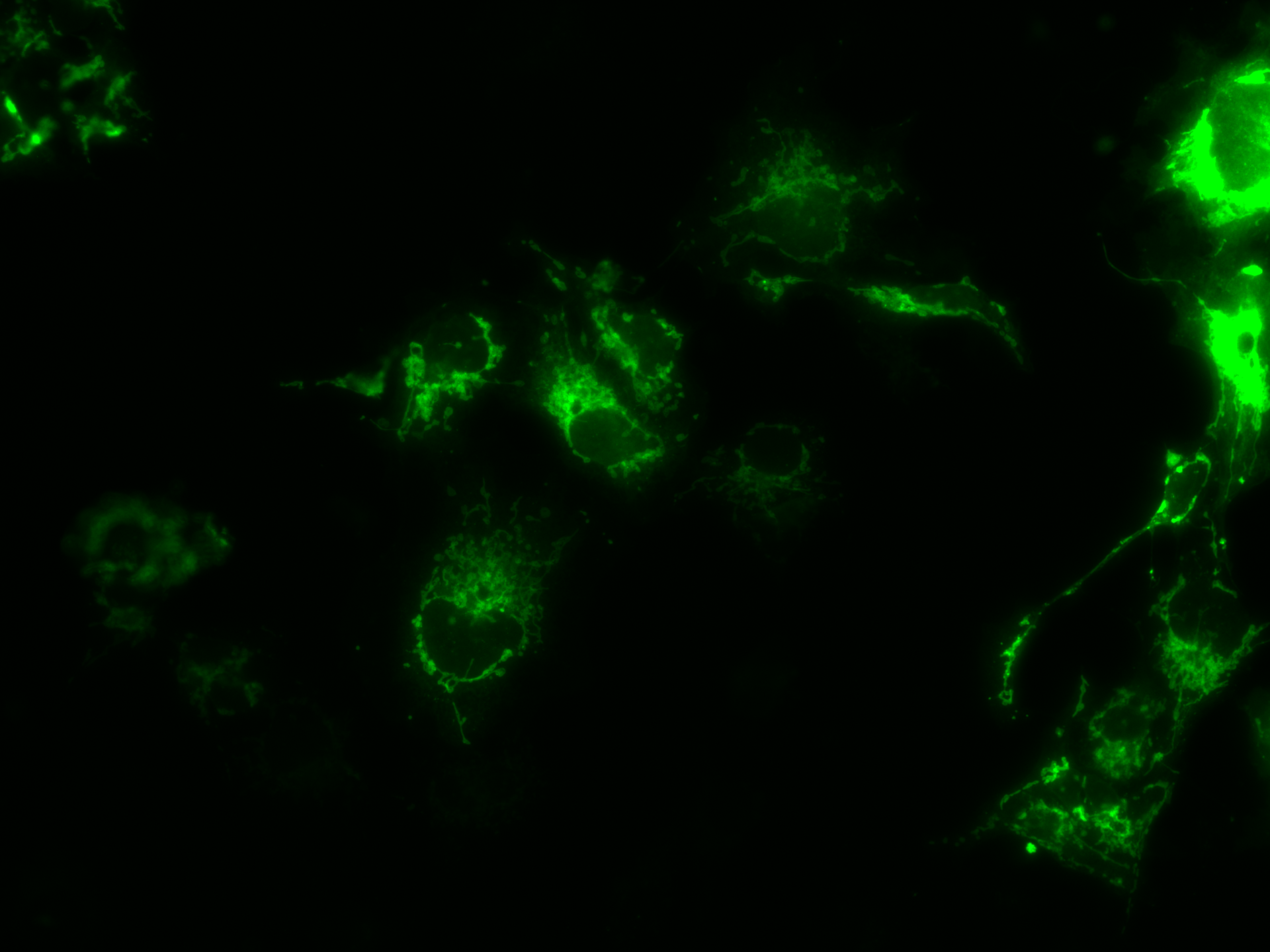

Supplement: S1 Fig — (TIF) [file pone.0191108.s001.tif]

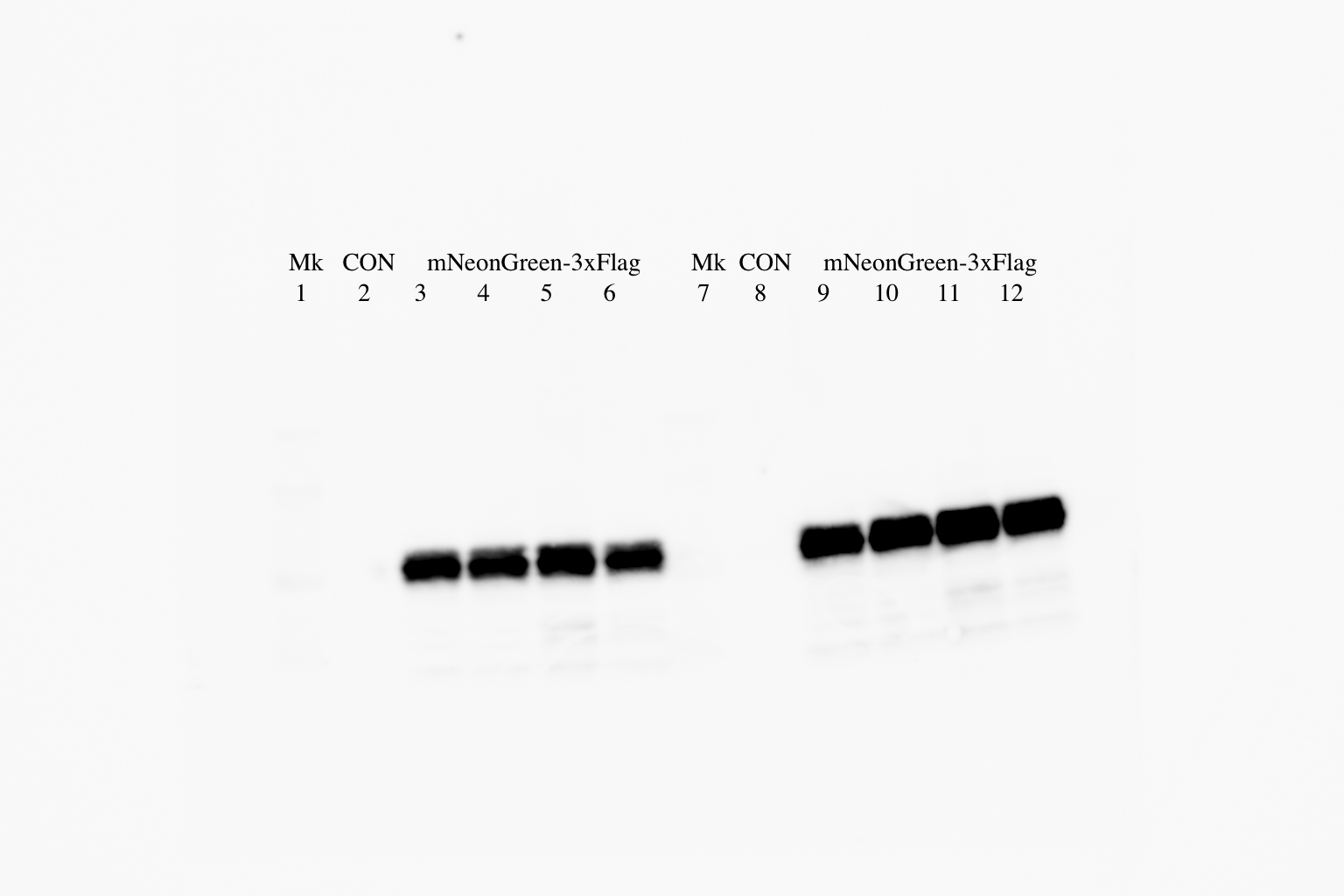

Supplement: S2 Fig — (A) The original immunoblotting data of Humanized mNeonGreen with 3xFlag tag using mouse monoclonal anti-Flag-M2 antibody. The lysate of cells transfected into pmNeonGreenHO-3xFLAG (mNeonGreenHO-3xFlag) or a control plasmid (CON) was separated on SDS-PAGE, and Flag-tagged proteins and GAPDH were recognized. From the left, molecular weight marker (Mk, lanes 1 and 7), negative control (CON) (lanes 2 and 8), mNeonGreen-3xFlag (lanes 3–6 and 9–12). (B) The original data of Humanized mNeonGreen with 3xFlag tag using a rabbit polyclonal anti-glyceraldehyde-3-phosphate dehydrogenase (GAPDH) antibody. As a loading control, GAPDH in the lysate was recognized after removing anti-Flag-M2 antibody. (TIFF) [file pone.0191108.s002.tiff]
